# Supplementary material for: P=O Functionalized Black Phosphorus/1T-WS2 Nanocomposite High Efficiency Hybrid Photocatalyst for Air/Water Pollutant Degradation
Source: Int J Mol Sci. 2022 Jan 10;23(2):733. doi: 10.3390/ijms23020733 (PMC8776125; doi:10.3390/ijms23020733)
Supplement: Supplementary file 1 [file ijms-23-00733-s001.zip › ijms-1534851-supplementary.pdf]

Supplementary Information for

# P=O Functionalized Black Phosphorus/1T-WS_2 Nanocomposite High Efficiency Hybrid Photocatalyst for Air/Water Pollutant Degradation

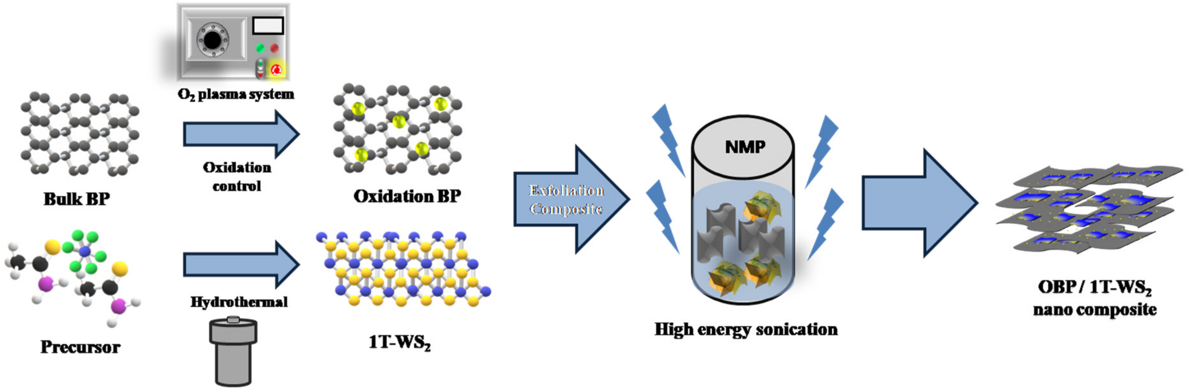

Scheme 1. Schematic illustration of the preparation of oxidation controlled black phosphorus /1T-WS_2 nanocomposite.

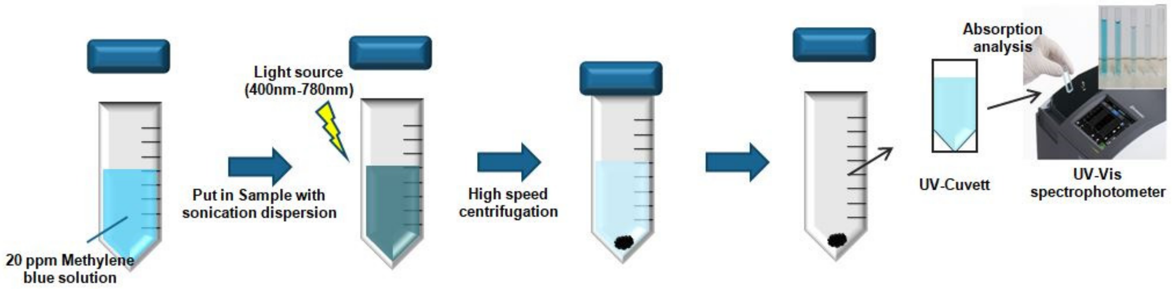

Scheme 2. Procedure for experiments regarding methylene blue solution degradation photocatalytic performance.

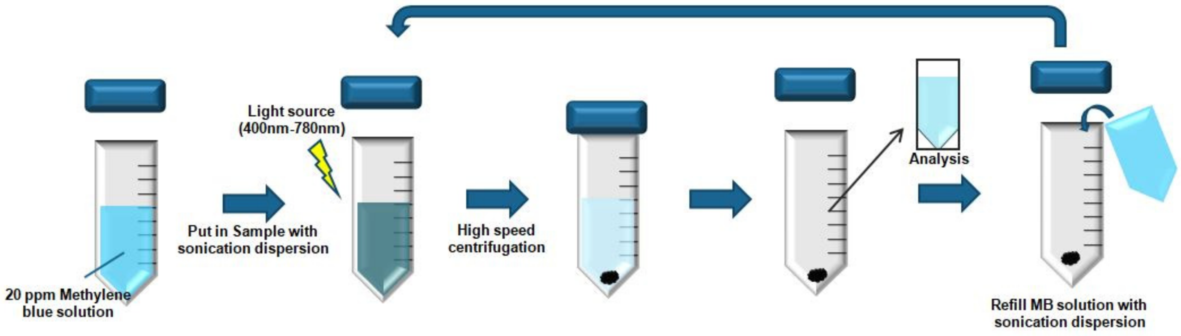

Scheme 3. Procedure for methylene blue degradation recycling test.

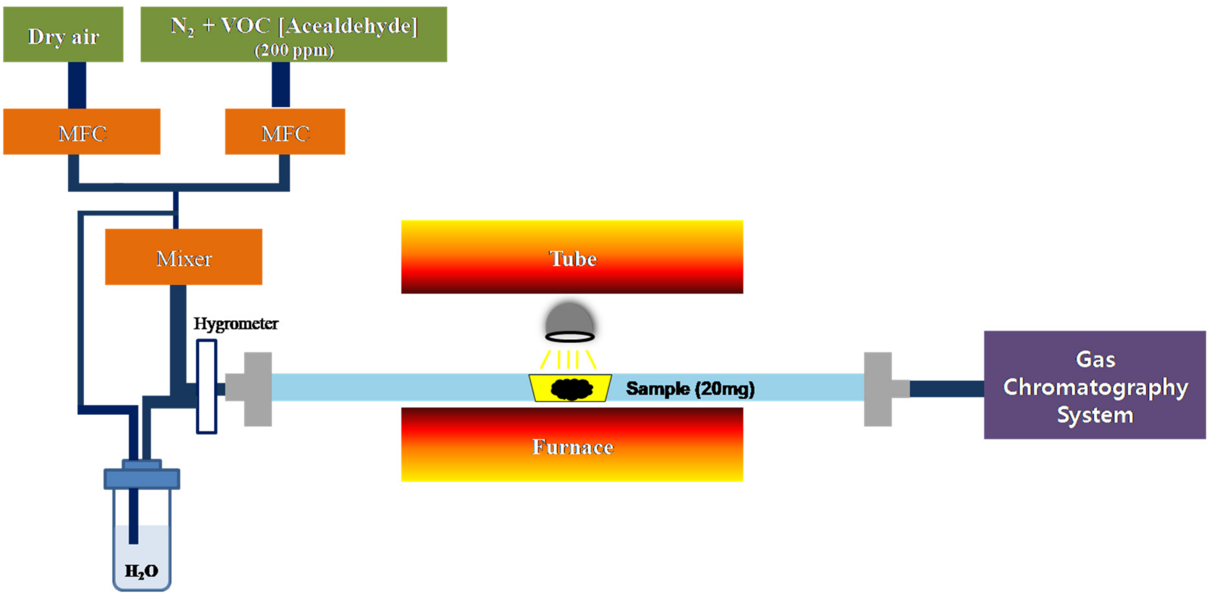

Scheme 4. Experimental set-up for photocatalytic VOC degradation.

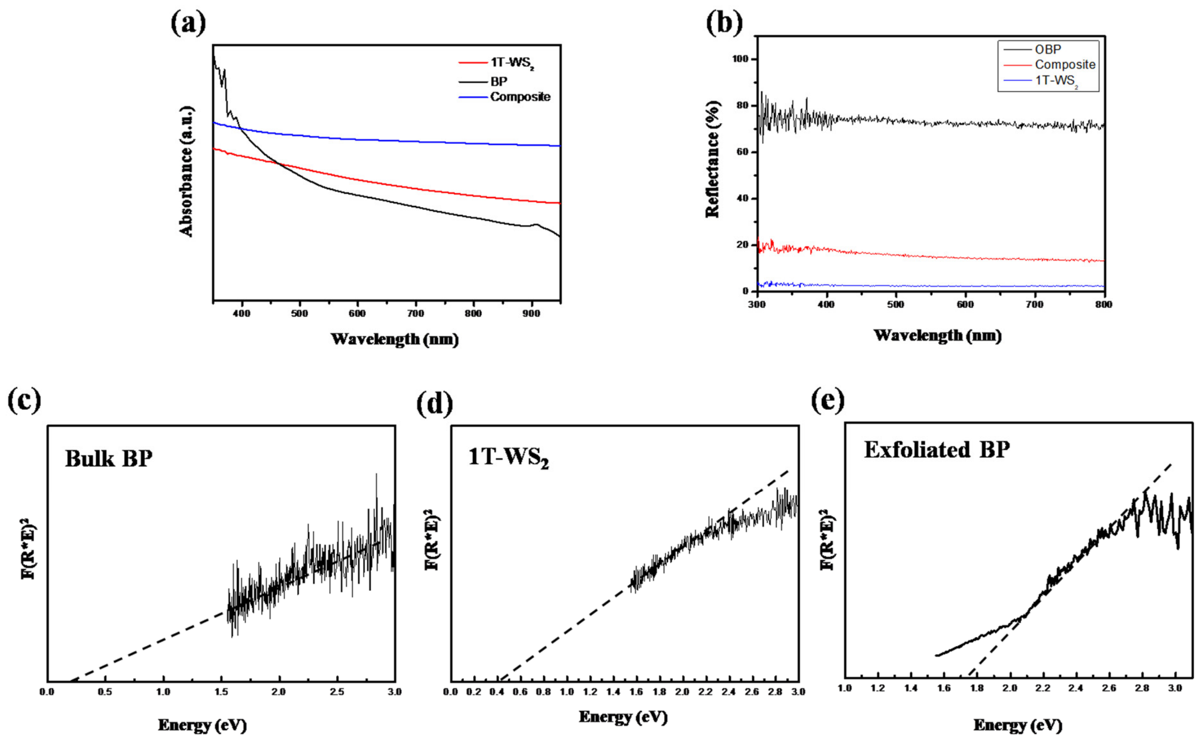

Figure S1. (**a**) Absorbance spectra of OBP, 1T-WS_2, and nanocomposite. (**b**) Diffuse reflectance spectra of OBP, 1T-WS_2, and nanocomposite.The bandgap of (**c**) OBP, (**d**) 1T-WS_2, and (e) exfoliated BP calculated using Kubelka–Munk'stheory.

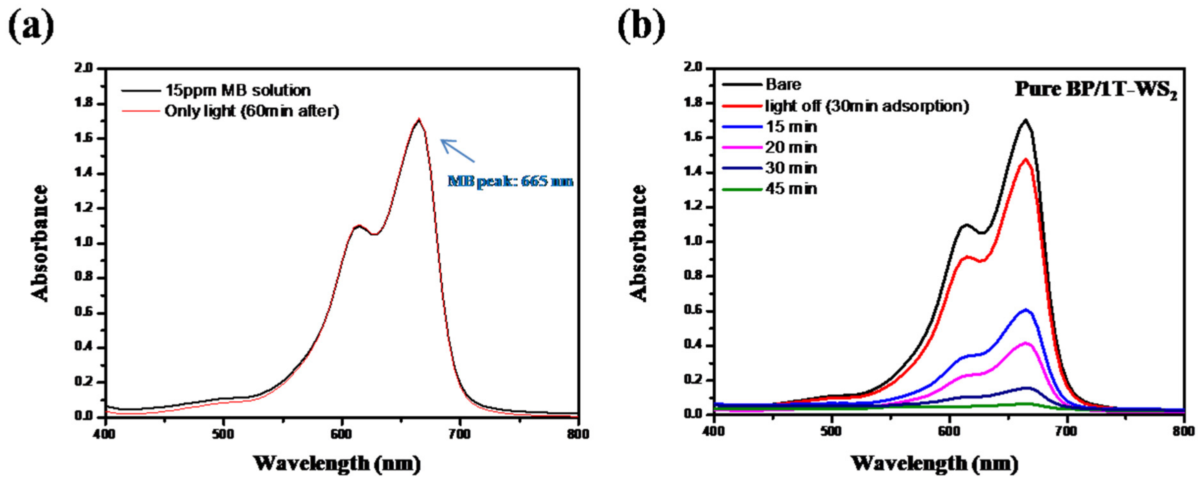

Figure S2. (**a**) Results of methylene blue solution irradiationfor 60 min under light without photocatalytic material. (**b**) UV-vis spectra of the methylene blue solution (20 ppm), which was degraded bypure BP/1T-WS_2.

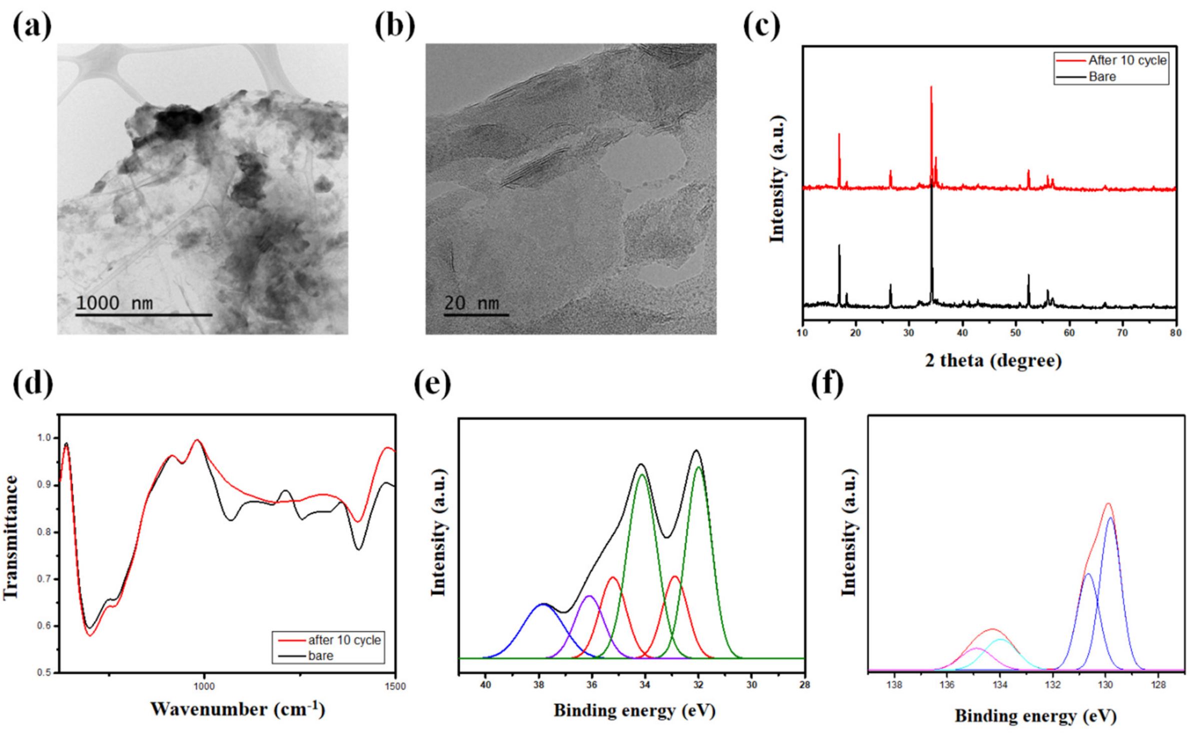

Figure S3. (**a**,**b**) TEM, (**c**) XRD, (**d**) FT-IR, and (**e**,**f**) XPS data of the OBP/1T-WS_2 nanocomposite after continuous photocatalytic reaction stability testing in Figure 6c.

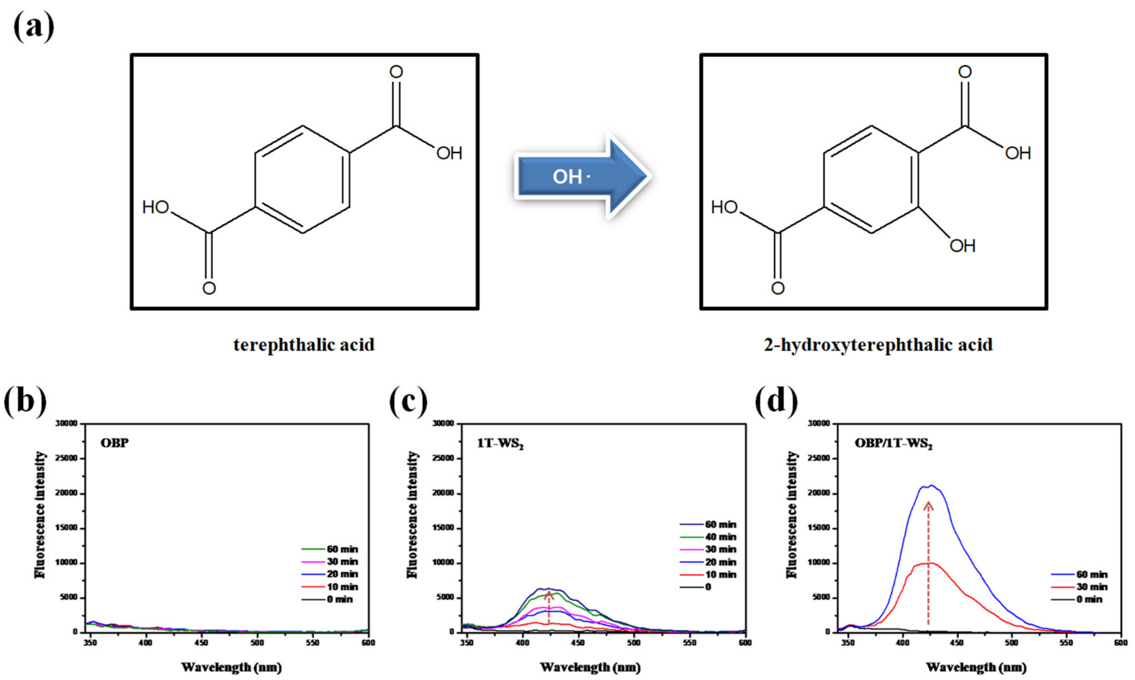

Figure S4. (**a**) The formation of hydroxy products due to the reaction between terephthalic acid and ·OH. The production of 2-hydroxyterephthalic acid was monitored by measuring the fluorescence emission spectra (λ_{em}= 425 nm, λ_{ex}= 315 nm) obtained over OBP (**b**), 1T-WS_2 (**c**), OBP/1T-WS_2 (**d**).

Table S1. Comparison with reported cases of MB decomposition visible photocatalyst.

Photocatalyst material	Bare MB Absorbance	Kinetic parameter	Reaction time	Recycle Test	Reference
CeO_2/TiO_2 nanotube	0.8	3.6 x 10^{-2} min^{-1}	150 min	3 cycle	79
Palladium doped TiO_2	3.0	4.4 x 10^{-2} min^{-1}	150 min	10 cycle	97
TiO_2 / Graphene Porous composite	10 mgL^{-1}	2.1 x 10^{-2} Min^{-1}	180 min	3 cycle	98
C-doped anatase TiO_2	Not shown / 50 ml	0.7 x 10^{-2} Min^{-1}	90~120 min	N/A	99
**OBP/1T-WS_2 nanocomposite**	**1.6 (20 mgL^{-1})**	**10.31 x 10^{-2} min^{-1}**	**20 min**	**10 cycle**	**This study**

Table S2. Comparison with reported cases of acetaldehyde decomposition visible photocatalyst.

Photocatalyst material	Light Source	catalytic efficiency	Reference
GO-TiO_2	200 W Xenon lamp	65% degradation 25 ppm / 0.1g	100
S-doped TiO_2	F8T5 WW 8W lamp	60% degradation 500mg 500 mL of acet-aldehyde with 500 μM initial concentration (in aqueous solution)	101
$Mica/TiO_2/Fe_2O_3$ composite	10 mgL^{-1}	80% degradation 500 ppm 8 sccm 0.1g	102
**OBP/1T-WS_2 nanocomposite**	**20W White LED**	**60 μmol / g min**	**This study**
